# Physical insights on transistors based on lateral heterostructures of monolayer and multilayer PtSe_2_ via Ab initio modelling of interfaces

**DOI:** 10.1038/s41598-021-98080-y

**Published:** 2021-09-16

**Authors:** Gaetano Calogero, Damiano Marian, Enrique G. Marin, Gianluca Fiori, Giuseppe Iannaccone

**Affiliations:** 1 Dipartimento di Ingegneria dell’Informazione, Università di Pisa, Via Girolamo Caruso 16, 56122 Pisa, Italy; 2 Consiglio Nazionale delle Ricerche, Istituto per le Microelettronica e Microsistemi, Z.I. VIII Strada 5, 95121 Catania, Italy; 3 Dipartimento Electronica, Facultad de Ciencias, Universidad de Granada, 18071 Granada, Spain

**Keywords:** Nanoscale devices, Two-dimensional materials

## Abstract

Lateral heterostructures (LH) of monolayer-multilayer regions of the same noble transition metal dichalcogenide, such as platinum diselenide (PtSe_2_), are promising options for the fabrication of efficient two-dimensional field-effect transistors (FETs), by exploiting the dependence of the energy gap on the number of layers and the intrinsically high quality of the heterojunctions. Key for future progress in this direction is understanding the effects of the physics of the lateral interfaces on far-from-equilibrium transport properties. In this work, a multi-scale approach to device simulation, capable to include ab-initio modelling of the interfaces in a computationally efficient way, is presented. As an application, p- and n-type monolayer-multilayer PtSe_2_ LH-FETs are investigated, considering design parameters such as channel length, number of layers and junction quality. The simulations suggest that such transistors can provide high performance in terms of subthreshold characteristics and switching behavior, and that a single channel device is not capable, even in the ballistic defectless limit, to satisfy the requirements of the semiconductor roadmap for the next decade, and that stacked channel devices would be required. It is shown how ab-initio modelling of interfaces provides a reliable physical description of charge displacements in their proximity, which can be crucial to correctly predict device transport properties, especially in presence of strong dipoles, mixed stoichiometries or imperfections.

## Introduction

A promising route for the next-generation of efficient field-effect transistors (FETs) is to exploit lateral heterostructures (LHs) of two-dimensional (2D) materials, such as transition metal dichalcogenides (TMDs)^1–3^. Platinum diselenide (PtSe_2_) and other noble TMDs are particularly appealing, because their energy gap depends on the number of layers, varying in a broad range from few eVs (semiconductor) to 0 eV (semimetal)^4–10^. Such tunability enables the fabrication of LH-FETs with high-quality, lattice-matched metal–semiconductor heterojunctions, which is crucial to obtain low-resistance contacts, required for both high performance and low power operation^2,11,12^. Inspired by well-established CMOS processing^13^, it has been recently proposed that by making the TMD channel thin under the gate but thick under the source and drain contacts—e.g. creating a recessed TMD channel^14,15^—it is possible to achieve efficient gate control while keeping contact resistance down to 350–400 Ω µm^14^. Similar improvements have been reported or predicted for LH-FETs based on PtSe_2_^7,14,15^, but also other TMDs^8,16^ and black phosphorus^17–20^. Engineering such nanoscale LH-FETs relies on the understanding and control of atomic-scale details of the lateral interfaces, which dominate the nearby charge distribution, thus altering the Schottky barrier shape and affecting the device performance^21^.

From the theoretical perspective, the problem of reliably simulating realistically-sized LH-FETs in far-from-equilibrium conditions is an open issue, especially when it comes to modelling charge displacements or non-idealities at interfaces^21,22^. In most cases, it requires tackling two major challenges. One is to identify a multi-scale procedure which, on the basis of ab-initio atom-by-atom simulations, can yield a small-sized Hamiltonian able to capture the electronic behaviour of the system within an energy window of interest, so that the most relevant transport features can be reproduced with acceptable computational cost. The second one is to include local dipoles and charge displacements around interfaces from the atomistic *ab-initio* structure into the mesh used within a device simulator to solve device electrostatics far from equilibrium. Despite its importance, the latter aspect is often overlooked and neglected in device simulations, which rely on determining free-charge redistribution throughout the device according to the solution of the Poisson equation on a grid that is often too coarse to enable the capture of charge redistribution at the interface. In fact, nowadays the ability of including such fine level of detail in a far-from-equilibrium device simulation inevitably comes with the price of a high computational cost or a relatively small device size.

State-of-the-art modelling approaches are based on projecting an Hamiltonian obtained with Density Functional Theory (DFT) onto a basis set of Maximally Localized Wannier Functions (MLWF)^23,24^ or Pseudo-Atomic Orbitals (PAO)^25^. The former, in particular, allows one to reproduce bands within a relevant energy window with very good accuracy. Both vertical and lateral 2D heterostructures have been studied exploiting such method^12,24,26,27^. For instance, LHs of semimetallic 1 T and semiconducting 2H phases of MoS_2_^26^, as well as LHs of multilayer-monolayer noble metal disulphides^12^ were investigated by using DFT to estimate the Schottky barrier height, and then using it to rigidly shift the Hamiltonians of the two junction components represented on a MLWF basis. Interactions at the interface were defined therein using a combination of the internal couplings of the bulk components. As a result, the total heterostructure Hamiltonian had a small dimension, as few Wannier Functions (≤ 16) were enough to reproduce the bands of the bulk components over a wide energy window. However, despite its computational advantage, so far this method has not been able to properly capture neither the charge displacements at the interfaces, occurring on a fine spatial scale, nor their finite width.

A direct “wannierization” (i.e. projection onto a MLWFs basis) of the whole heterostructure system has been recently adopted by Szabó et al*.*^24,27^ who used it to study both weakly and strongly coupled 2D heterojunctions, such as TMD-TMD and metal-TMD cases, respectively, directly extracting the Hamiltonian of all the heterostructure sub-regions from the heterostructure MLWF Hamiltonian. Compared to the approach described in Ref.^26^, in this case the interface regions can be reliably represented in real space, with the same basis as that of the individual bulk components. However, the dimension of the device Hamiltonian, and hence the computational burden of the calculations, is much larger, because the number of Wannier Functions needed to model an heterostructure is higher.

In this work we present a very efficient multi-scale procedure that enables the simulation of far-from equilibrium transport in realistically sized LH-FETs, accounting for the effects of local charge displacements at the lateral interfaces at low computational cost. We demonstrate the usefulness and the versatility of the procedure by investigating the performance potential of mono-multilayer PtSe_2_ LH-FETs with different channel length, lead thickness and junction smoothness, considering the requirements set out in the 2020 edition of the International Roadmap for Devices and Systems (IRDS)^28^. In particular, we emphasize how *ab-initio* modelling of interfaces and a reliable physical description of charge distribution in their proximity are crucial to correctly predict charge transport in LH-FETs.

In order to successfully include such fine charge displacements in the coarse electrostatic grid, we propose to directly include their effect on the potential as a fixed contribution. This is equivalent to neglect variations of charge distribution at the interface occurring on a length scale much smaller than the minimum spacing of the electrostatic grid, as it is typical of mean field treatments. Therefore, we extract the on-site energy along the LH from *ab-initio* DFT simulations, and then map it directly onto the modelled device.

We anticipate that the scope of this work is not to perfectly model an interface, but rather to provide a modular Hamiltonian enabling one to explore several devices, materials or geometries capturing the relevant physics at a reasonably low computational cost. For a deeper chemical understanding of how the junctions atomic-scale details, such as defects, functionalization, or roughness, affect the heterostructure electronic properties, a more accurate orbital-resolved and entirely DFT-based transport code (e.g., Transiesta^29^) would better be used on a preliminary level, even though on relatively smaller devices and in near-to-equilibrium conditions.

The article is organized in two parts. We first introduce, step by step, our multi-scale procedure in the context of building a model for bilayer-monolayer LH-FET of PtSe_2_. We then apply this method to predict the performance of both bilayer-monolayer and four-layer-monolayer PtSe_2_ LH-FETs, with sharp and gradual junctions, in far-from-equilibrium conditions.

## Results and discussion

### Overview of the multi-scale model

**Figure 1:**
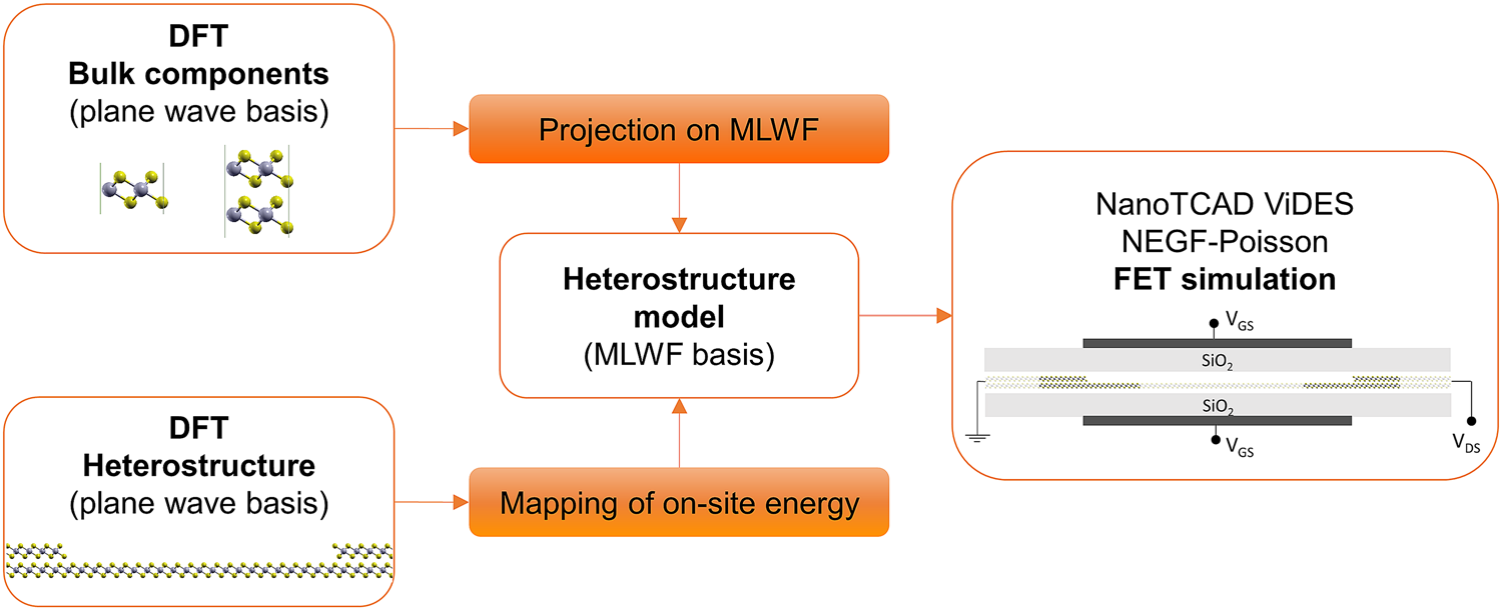
Block diagram of the multi-scale procedure. Bulk DFT calculations of the materials forming the LH are performed using a plane wave basis. The resulting Hamiltonians are then projected onto MLWF and used as building blocks to construct a LH Hamiltonian with an arbitrarily long channel. Then a model of the LH is simulated with DFT and the on-site energy profile, *E*_*on-site*_, in the direction normal to the interface is extracted. This energy profile is directly mapped on the diagonal of the MLWF LH Hamiltonian, thus correctly capturing the DFT band alignment across the LH and the electrostatic effects due to charge redistribution around lateral interfaces. The resulting LH Hamiltonian is finally used as input in NanoTCAD ViDES to simulate LH-FETs in far-from-equilibrium conditions. This figure was made using VMD [v1.9.1, https://www.ks.uiuc.edu/Research/vmd/vmd-1.9.1].

We propose in this work a multi-scale modelling approach consisting in three steps, schematically illustrated in Fig. 1. First, we perform plane-wave DFT simulations of the bulk components of the LH, from which we extract a real-space small-sized Hamiltonian in the MLWF basis. Second, we use DFT to model the LH and calculate the on-site energy profile *E*_*on-site*_ (*x*) along the transport direction *x*. Third, we construct a larger-scale device Hamiltonian in the MLWF basis by coupling the component Hamiltonians together, taking care of mapping site-by-site the *ab-initio* variation of *E*_*on-site*_ in proximity of the junctions. As will be extensively discussed below, this will ensure that the model reproduces the effects of relevant local charge variations. Finally, the MLWF device Hamiltonian is used within the open-source device simulator NanoTCAD ViDES^30^ to simulate ballistic transport in equilibrium and far-from-equilibrium conditions. In the next sections, we go through each of the steps towards the construction of a model for bilayer (2L)-monolayer (1L) PtSe_2_ LH-FET.

### Ab-initio models of bulk 2L and 1L PtSe_2_

All our DFT calculations are performed using the Quantum Espresso electronic structure package^31^ sampling the first Brillouin zone with a 8 × 12 × 1 Monkhorst–Pack grid (increased to 16 × 24 × 1 for computing the DOS). We adopt ultra-soft pseudopotentials, the Perdew-Burke-Ernzerhof exchange–correlation functional^32^ with D2 van der Waals correction^33^, an energy cutoff of 40 Ry and a charge-density cutoff of 400 Ry. We avoid spurious interactions between periodic replicas along the out-of-plane direction *z* by introducing 3 nm of vacuum above the top-most layer of PtSe_2_ in the unit cells. A dipole correction is also used to avoid spurious electric fields along *z*, using the method described in Ref.^34^ and implemented in Quantum Espresso.

**Figure 2:**
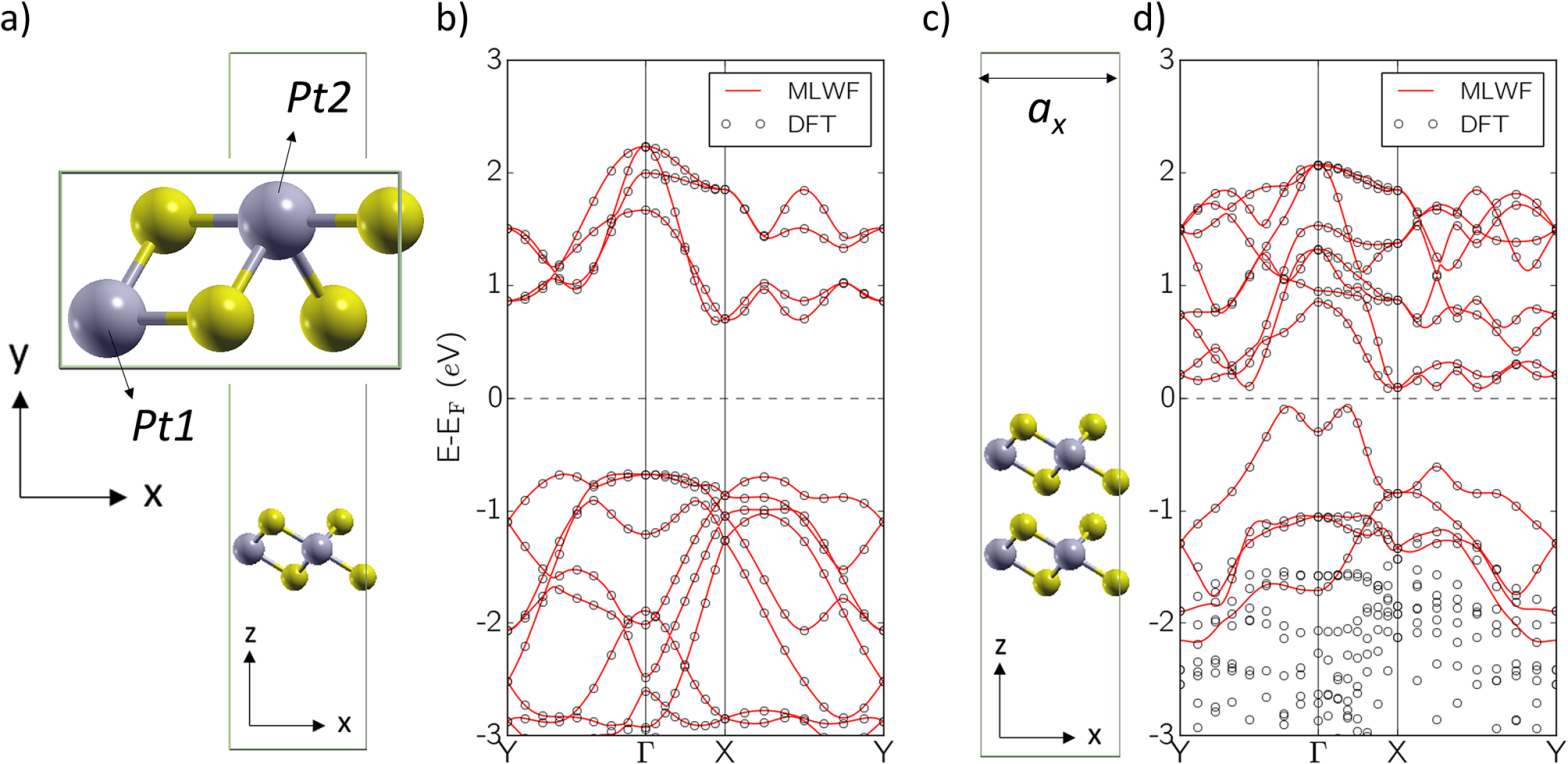
DFT models. (**a**) Unit cell and (**b**) DFT and Wannier interpolated bands for bulk 1L PtSe_2_, with Pt in grey and Se in yellow. The inset shows the cell from the top. (**c**) Unit cell and (**d**) DFT and Wannier interpolated bands for bulk 2L PtSe_2_. This figure was made using VMD [v1.9.1, https://www.ks.uiuc.edu/Research/vmd/vmd-1.9.1] and Matplotlib [v3.3.3, https://doi.org/10.5281/zenodo.4268928].

We describe PtSe_2_ using an orthorhombic Bravais lattice. We simulate the 1 T phase and use AA stacking to model multilayer structures, as they were reported to be the most stable configurations^6,35^. We optimize the lattices by relaxing atomic coordinates self-consistently until all residual forces acting on each atom are below 10^–3^ Ry Bohr^−1^. At equilibrium we find a cell length *a*_*x*_ = 0.65 nm, an in-plane nearest Pt–Pt distance of 0.37 nm, an interlayer Pt–Pt distance of around 0.47 nm and around 0.26 nm layer thickness (Se-Se distance), in good agreement with experimental and calculated values available in literature^7,35,36^. The geometries and band-structures for both 1L and 2L systems are shown in Fig. 2. We extract a band-gap of 1.355 eV for the 1L case and a band-gap of 0.15 eV for the 2L case, in reasonable agreement with experiments and other DFT studies^7,35,37,38^. We remark that the bandgap energy found by DFT is underestimated, and that a GW correction would most likely provide larger values. This would have the beneficial effect of reducing intra-band tunneling effects when considering short channel devices.

Then, we use the open-source code Wannier90^39^ to generate a Hamiltonian for the 1L and 2L systems in a basis set of MLWFs, using the same *k*-points grid of the DFT calculations. For both cases we project the plane-wave Hamiltonian onto N_w_ = 12 Wannier Functions to reproduce the bands shown in red in Fig. 2b,d, which span a range of over 4 eV around the mid-gap level.

### Multi-scale construction of MLWF device Hamiltonian

**Figure 3:**
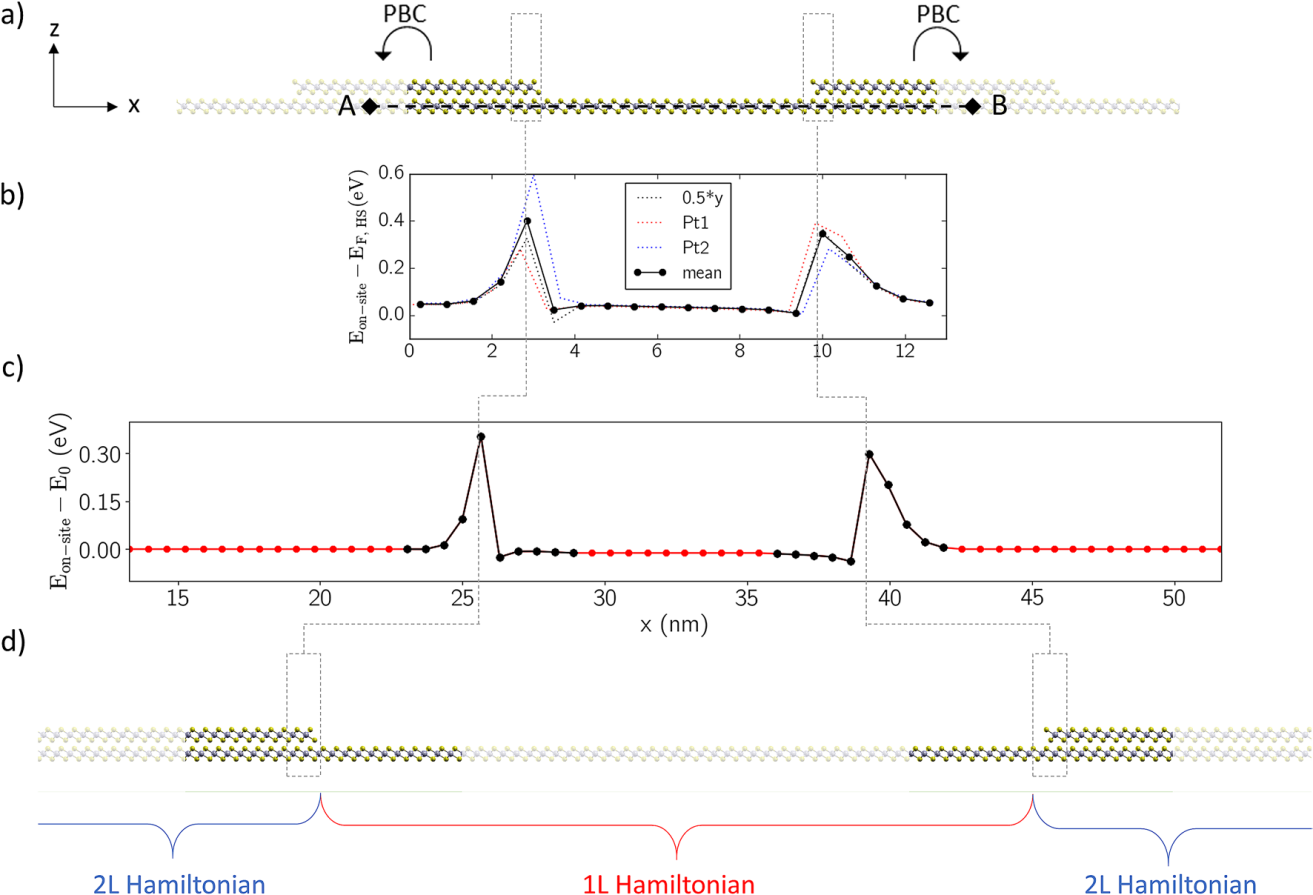
Multi-scale approach to construct the Hamiltonian for a long transistor channel. (**a**) Supercell used to model the heterostructure in DFT, with periodic boundary conditions (PBC) highlighted. (**b**) On-site energy profile *E*_*on-site*_ along the black dashed line in (**a**), aligned w.r.t. the HS Fermi level *E*_*F,*__*HS*_. Dotted lines correspond to different *y* coordinates of the line, namely those of the Pt atoms indicated in Fig. 2a or the midpoint between them. The average of these curves is shown in solid black. The distance between points along *x* is *a*_*x*_. (**c**) Profile of *E*_*on-site*_ along a LH constructed by elongating 1L and 2L regions and using the potential from panel (**b**) around the interfaces (black), plotted w.r.t. *E*_*0*_ = *E*_*on-site*_(*x* = 0), i.e., the left-most value in panel (**b**). The potential elsewhere (in red) is fixed to that of the bulk-most sites in the 2L (1L) regions. (**d**) Geometry of the final device, highlighting the regions in which the 1L (2L) MLWF Hamiltonians have been used. Dotted lines are drawn to guide the eye throughout the multi-scale procedure. This figure was made using VMD [v1.9.1, https://www.ks.uiuc.edu/Research/vmd/vmd-1.9.1] and Matplotlib [v3.3.3, https://doi.org/10.5281/zenodo.4268928].

We build the geometry for the 2L-1L LH of PtSe_2_ by embedding 10 cells of 1L PtSe_2_, tiled along *x*, between 2L regions, as illustrated in Fig. 3a. The obtained supercell, periodic along both *x* and *y* directions, contains a 6.4 nm long 1L region. We terminate the top layer at both interfaces with Se atoms, in order to minimize possible in-plane electric fields across the 1L region, and we check that the positions of all edge Se atoms are only slightly modified upon optimization of the geometry close to the interfaces (all residual forces were below 10^–3^ Ry Bohr^–1^). From the DFT-calculated ground state of the LH we compute the on-site energy profile *E*_*on-site*_ (*x*) ≡ *E*_*midgap*__,__*HS*_ (*x*) along the transport direction *x*. This is done by following the procedure reported by Cusati et al.^40^, which gives:

1 $$E_{on - site} (x) = E_{midgap,\,bulk} (x) + \left[ {V_{ref,\,HS} (x) - V_{ref,\, bulk} (x)} \right],$$

where *E*_*midgap,*_ _*bulk*_ (*x*) is the midgap energy in the isolated bulk sub-systems. The electrostatic potential *V*_*ref,*_ _*HS*_ (*x*) is obtained by summing the bare and the Hartree potential contributions, expressed in eV, on a line along *x* within the LH supercell at fixed (*z*_*ref*_*,* *y*_*ref*_) coordinates, and then extracting the value of the potential at a reference point in every interval [*m* × *a*_*x*_, (*m* + 1) × *a*_*x*_] with *a*_*x*_ being the unit cell length along *x*, *m* = 0, … *M*, with *M* an integer and *M* × *a*_*x*_ being the total size of the supercell along *x*. Likewise, *V*_*ref,*_ _*bulk*_ (*x*) is the electrostatic potential extracted at the same reference point, but in the bulk 1L and 2L unit cells.

The profile of *E*_*on-site*_ (*x*) for the 2L-1L PtSe_2_ LH is plotted in Fig. 3b, with reference to the LH Fermi level *E*_*F,*__*HS.*_ This is obtained by extracting the electrostatic potential along the black A-B line indicated in Fig. 3a, with *z*_*ref*_ fixed as the coordinate of the Pt atoms in the lowest PtSe_2_ layer, and *y*_*ref*_ fixed at three different values, namely the *y* coordinate of the Pt atoms labelled “*Pt1*” and “*Pt2*” in Fig. 2a, and their mid-point. Their average is taken as final reference on-site energy profile *E*_*on-site*_ (*x*) in the LH and is shown as a black solid line in Fig. 3b. As can be seen, *E*_*on-site*_ shows a ~ 0.4 eV variation in the 2L regions next to the interfaces, as a direct consequence of charge redistribution. The slight deviation from zero observed in the 2L regions, far from the junctions, is due to the fact that *E*_*F,HS*_ is not exactly localized in the 2L midgap level.

The profile *E*_*on-site*_ and the bulk MLWF Hamiltonians for the 2L and 1L systems introduced in the previous section are finally used as input to generate the MLWF Hamiltonian for a LH-FET structure with longer 2L and 1L regions, as schematically illustrated in Fig. 3c,d. The procedure for this Hamiltonian construction consists in two steps. The first step is to connect the MLWF Hamiltonians of the bulk components and is based on the method reported in Refs.^23,26^. This step yields a MLWF Hamiltonian for the LH in block-tridiagonal form, with arbitrary length for the 1L and 2L regions and, most importantly, a very small size (see Supplementary Information). The second step consists in mapping *E*_*on-site*_ onto the longer LH. In particular, Fig. 3c shows how *E*_*on-site*_ is mapped site by site in proximity of the junctions, and how *E*_*on-site*_ is fixed to a constant—equal to its average central (extreme) values—in the sites belonging to the extended 1L (2L) regions, indicated in red in Fig. 3c.

We anticipate here that the variation of *E*_*on-site*_ close to an interface is an electrostatic effect due to charge redistribution in its proximity, in turn occurring in response to the junction chemical environment and the band alignment between the interface components. As will be pointed out in the next section, by “freezing” this variation in the static part of the Hamiltonian, and letting the free charge in the system self-consistently adjust boundary conditions of the potential in a device simulator such as NanoTCAD ViDES^30^, we can reproduce with reasonable accuracy the effects of interface atomic details on transport. The main approximation made here is the one typical of mean field approaches, i.e., that we can neglect charge profile variations on a length scale much smaller than the spacing of the grid used for electrostatics in the considered bias voltage range.

### Electrostatics in 2L-1L PtSe_2_ LH-FET including ab-initio interface modelling

In this section we present how *ab-initio* interface modelling (“AbInIM” for short) affects the results of transport simulations in the context of a LH-FET based on the 2L-1L PtSe_2_ heterostructure discussed so far.

**Figure 4:**
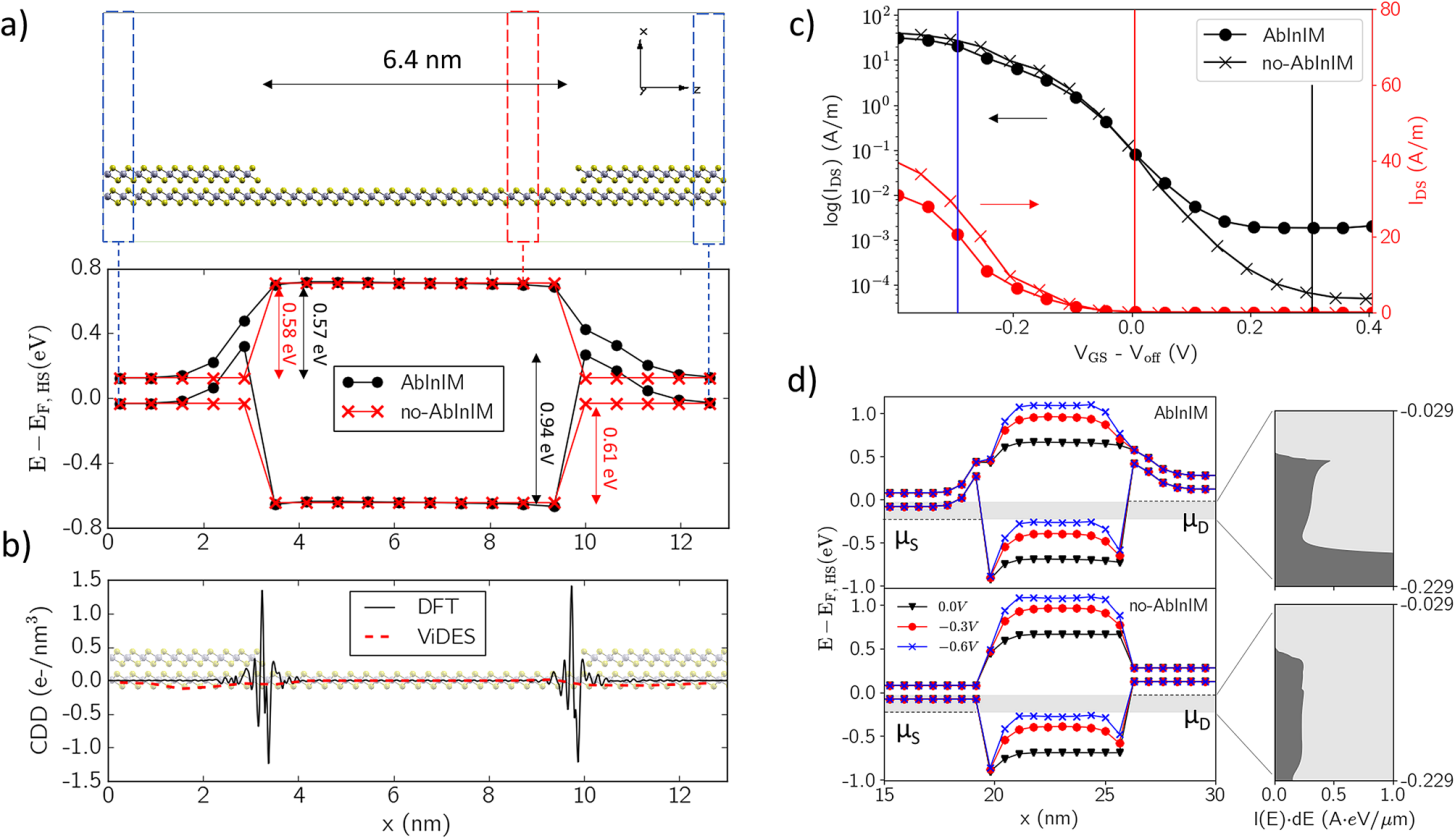
Charge transport with and without mapping *E*_*on-site*_ from DFT. (**a**) Top: supercell used to model the 2L-1L LH of PtSe_2_. Bottom: Conduction and valence band edges across the LH, obtained mapping (black) the full DFT potential or simply aligning according to 1L and 2L PtSe_2_ electron affinity (red). The barrier heights for electrons and holes are highlighted. (**b**) Charge density difference, averaged over *yz* planes, from DFT (black solid) and from self-consistent NEGF-electrostatics simulations (red dashed). The DFT supercell is also shown in background. (**c**) Current–voltage characteristics for the PtSe_2_ LH-FET at *V*_*DS*_ = -0.2 V in linear (red) and semi-logarithmic (black) scale, with *V*_*off*_ = -0.306 V (-0.294 V) for the “AbInIM” (“no-AbInIM”) case. d) Band diagrams at *V*_*GS*_ = 0.0 V, -0.3 V and -0.6 V, indicated with vertical lines in panel (**c**), for the two approaches. Panels on the right side show the spectral current within the bias window in the two cases, at *V*_*GS*_ = 0.0 V. This figure was made using VMD [v1.9.1, https://www.ks.uiuc.edu/Research/vmd/vmd-1.9.1] and Matplotlib [v3.3.3, https://doi.org/10.5281/zenodo.4268928].

From the on-site energy *E*_*on-site*_ in Fig. 3b one can draw the position of conduction and valence band edges across the LH. This is shown in Fig. 4a, in comparison with the case where mapping from DFT is carried out solely by considering differences in electron affinity (which we will refer to as “no-AbInIM” case for simplicity). From this figure one can immediately observe that the two profiles present some significant differences around the junctions, which reflect the band distortion and consequent charge redistribution naturally occurring when 2L and 1L PtSe_2_ regions are in contact. While the energy barrier for electrons is not too sensitive to ab-initio interface modelling, the shape and height of the barrier for holes is significantly different. While in the “no-AbInIM” case the junction is perfectly sharp, in the “AbInIM” case its effective width is found to be roughly 2 nm, with most of the potential variation occurring in the 2L region. For electrons, we find a very close value of *ΔE*_*e*_ ~ 0.57 eV and ~ 0.58 eV for the barrier height in the two cases, whereas for holes we obtain *ΔE*_*e*_ ~ 0.61 eV in the “no-AbInIM” case and a remarkably higher value of ~ 0.93 eV in the “AbInIM” case, when fully mapping *E*_*on-site*_. This is due to the upward distortion of the valence band in the 2L part of the junction, which leads to the accumulation of holes whenever the chemical potential of the 2L system lies in its valence band. Therefore, important quantitative differences between the two approaches are expected when studying transport in a p-type LH-FET.

Based on these considerations, we studied a 2L-1L-2L PtSe_2_ p-type LH-FET in the NanoTCAD ViDES NEGF-Poisson solver, with semi-infinite 2L regions embedding a 6.4 nm long 1L region (same as in DFT). These calculations were performed by fixing the valence band edge of the source and drain 50 meV above the electrochemical potential of source and drain, respectively, which corresponds to ensure charge neutrality for acceptor doping of 4 × 10^12^ cm^-2^ of the 2L source and drain (e.g., see Supplementary Figure S1)^12,23,26,30^.

In order to demonstrate that including the *E*_*on-site*_ profile in the NEGF Hamiltonian is reliable, we compare in Fig. 4b the charge density difference (CDD) obtained with DFT for the 2L-1L LH (solid black line) and that obtained with NanoTCAD ViDES (dashed red line) by self-consistently solving the electrostatics at equilibrium without external bias (i.e., no gate or supply voltages are applied), and mapping *E*_*on-site*_ as explained in the previous section. In DFT the definition of CDD is the standard $\rho (x) = S^{ - 1} \iint {dydz\left[ {\rho_{LH} (x,y,z) - \rho_{1L} (x,y,z) - \rho_{2L} (x,y,z)} \right]},$ where *S* is the *yz* area of the LH supercell, *ρ*_*LH*_ is the charge density of the LH and *ρ*_*1L*_ (*ρ*_*2L*_) is that of the isolated 1L (2L) region in the LH supercell. In NanoTCAD ViDES the definition is the same, but with *ρ*_*LH*_ being the free charge profile per unit volume obtained as a result of the simulation and *ρ*_*1L*_ (*ρ*_*2L*_) being the average free charge per unit volume in the 1L (2L) region farthest from the junctions. The DFT CDD clearly shows charge displacements in proximity of the interfaces, stronger around the 1L sites, with slightly different features between left and right ones, which in fact have slightly different atomic structures (see, e.g., Fig. 4a). These occur in a length scale smaller than inter-site distances, which represent the smaller steps of the device simulation grid. Hence, a self-consistent NEGF-electrostatic simulation would not be able to reproduce them. Indeed, it is for this reason that we propose to directly include the effect of charge redistribution on the potential, through the extraction procedure of the *E*_*on-site*_, as a fixed contribution (which is equivalent to “freeze” the charge displacements). Let us stress the fact that, consequently, such displacements do not appear in the NEGF-electrostatic calculation of mobile charge. Indeed, considering Fig. 4b, we see that CDD variations obtained from NEGF-electrostatics simulations are much smaller than those obtained with DFT. This is because all electrostatic contributions, that usually determine the charge density profile, are already contained inside the Hamiltonian, through *E*_*on-site*_, so that no additional free charge redistributions need to take place in the self-consistent electrostatic calculations.

### Charge transport in 2L-1L PtSe_2_ LH-FET including ab-initio interface modelling

In order to compare transport with and without *ab-initio* interface modelling in far-from-equilibrium conditions, we place two gate regions above and below the 1L region, with 0.5 nm equivalent oxide thickness (i.e. 0.5 nm of SiO_2_), and computed the transfer characteristics for an applied drain-to-source voltage *V*_*DS*_ = − 0.2 V, assuming fully coherent ballistic transport, acceptor doping the leads with 6 × 10^12^ cm^−2^, and setting the valence band edge 0.15 eV above the Fermi level in order to and ensure charge neutrality far from the junctions (see Supplementary Figure S1). The resulting *I*_*DS*_ − *V*_*GS*_ curves are plotted in Fig. 4c both in linear and semi-logarithmic scale. Here we call *V*_*off*_ the value of *V*_*GS*_ at which current is *I*_*off*_ = 10^–1^ A m^−1^ and we extract *I*_*on*_ at *V*_*GS*_ = *V*_*off*_ + *V*_*DS*_.

From the comparison of the two curves we can draw two important conclusions:

1. Both the subthreshold swing (SS; defined as the inverse slope of the *I*_*DS*_* − V*_*GS*_ curve in semilogarithmic scale in the subthreshold regime) and the *I*_*on*_*/I*_*off*_ ratio are worse in the “AbInIM” case. In particular, we find a SS of 77 mV dec^−1^ with a *I*_*on*_*/I*_*off*_ ratio of 6.8 × 10^2^ for the “AbInIM” case, and a SS of 66 mV dec^−1^ with a *I*_*on*_*/I*_*off*_ ratio of 9.3 × 10^2^ for the “no-AbInIM” case. This is a direct consequence of the higher barrier for the “AbInIM” case, already discussed in Fig. 4a and still observed in the far-from-equilibrium band diagrams of Fig. 4d. In addition to this, current on the ON state is further suppressed by the holes accumulating at the interfaces and screening other carriers propagating across the device.
2. In the subthreshold regime a higher tunnelling current is present in the “AbInIM” case. This has the important consequence of revealing the unsuitability of this device for low-power applications, which would not be possible with a “no-AbInIM” approach, where currents can reach values as low as 10^–4^ A m^−1^. The origin of this effect can be related to the valence band edge in the 2L part of the junction being bent upward in the “AbInIM” case, which causes accumulation of carriers and a consequent increase in tunnelling within the bias window. Such observation is confirmed by the spectral currents at *V*_*g*_ = 0 V plotted on the right side of Fig. 4d, where the large difference between the two cases is evident.

Finally, using the “AbInIM” approach presented above, we also assessed the performance of p-type (n-type) 2L-1L PtSe_2_ LH- FETs at larger supply voltage *V*_*DS*_ = − 0.5 V (+ 0.5 V), which is a more relevant operational condition in the light of the 2020 edition of the IRDS^28^. According to the consensus therein, future logic devices will need to be optimized towards either high-performance (HP) or low-power/high-density (HD) applications. The current in the off state must satisfy *I*_*off*_ = 10^–2^ A m^−1^ for HP applications, while a lower *I*_*off*_ is required, *I*_*off*_ = 10^–4^ A m^−1^, for HD applications. In each case, we define *V*_*off*_ as the gate voltage for which *I*_*DS*_ = *I*_*off*_.

We consider two different channel lengths, namely 6.4 nm and 12.8 nm. We fix the valence (conduction) band edge for the p- (n-) type FETs, 0.05 eV above (0.15 eV below) the electrochemical potential in the source and drain, which provides charge neutrality far from the heterointerfaces for a souce and drain acceptor doping of 4 × 10^12^ cm^−2^ (donor doping of 3 × 10^13^ cm^−2^). The obtained transfer characteristics for all cases are reported in Supplementary Figure S2, and the SS and *I*_*on*_*/I*_*off*_ ratio estimated for HP and HD digital applications are reported in Table 1. We find that the considered LH-FETs can reach very good SS in the range 61–72 mV dec^−1^ and ratios *I*_*on*_*/I*_*off*_ > 10^4^ for the HP optimized cases, which increases above 10^5^ in the HD ones. The longer channel benefits from lower SS, near the optimum 60 mV dec^−1^, enabling for HD applications, contrary to the shorter channel, where tunnelling yields a relatively large current in the sub-threshold regime. We also find no significant differences in performance between p- and n-type FETs for HP applications, whereas the p-type FET clearly outperforms the n-type for HD applications, yielding almost an order of magnitude improvement for both SS and *I*_*on*_/*I*_*off*_ ratio.

**Table 1:** Figures of merit for 2L-1L PtSe_2_ FETs. All figures of merit were obtained by mapping *E*_*on-site*_ from DFT. The IV curves from which the data were extracted are available in Supplementary Figure S2.

	V_DS_ [V]	L [nm]	High-performance		Low-power/High-density	
			SS [mV dec^−1^ ]	I_on_/I_off_	SS [mV dec^−1^]	I_on_/I_off_
2L-1L-2L p-type	− 0.5	6.4	71	1.3 × 10^4^	–	–
		12.8	68	1.4 × 10^4^	61	8.6 × 10^5^
2L-1L-2L n-type	+ 0.5	6.4	72	3.8 × 10^4^	–	–
		12.8	65	3.7 × 10^4^	66	1.6 × 10^6^

### Charge transport in 4L-1L PtSe_2_ LH-FET with sharp and smooth interfaces

**Figure 5:**
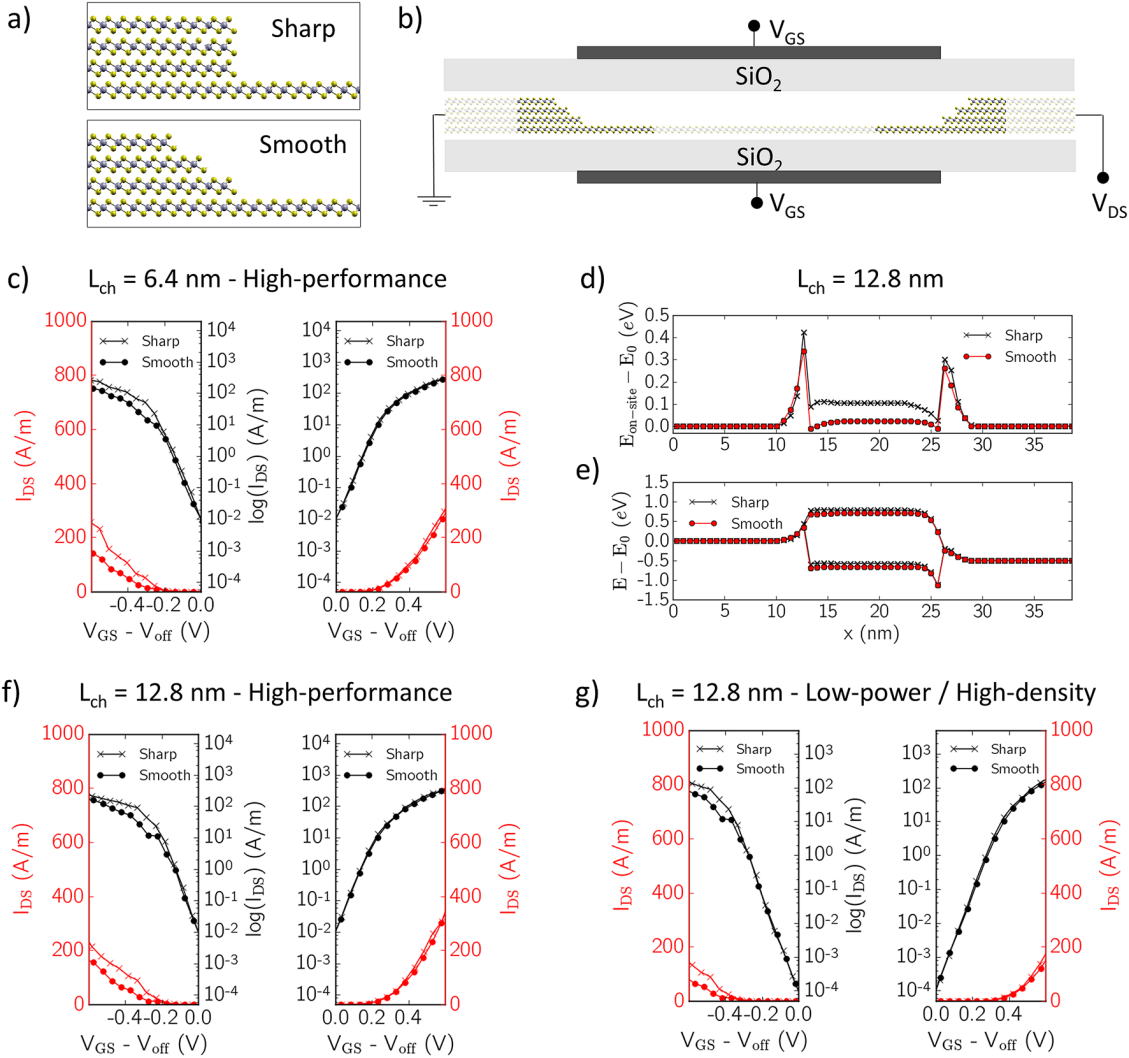
Far-from-equilibrium transport simulations of 4L-1L PtSe_2_ heterostructures. (**a**) Lateral (*xz*) view of sharp and 3-nm smooth interfaces. (**b**) double-gate FET configuration. (**c**) Transfer characteristics for a 6.4 nm long 1L channel in a FET with 4L leads and sharp (crosses) or smooth (circles) interfaces, optimized for HP processes. Red curves are in linear scale and black curves are in semilogarithmic scale. The n- (p-) branch is shown on the right (left). (**d**) On-site energy *E*_*on-site*_ along the heterostructures with sharp (black) and smooth (red) interfaces between the 4L leads and a 12.8 nm long 1L channel. *E*_*0*_ = *E*_*on-site*_(*x* = 0)*.* (**e**) Valence and conduction band diagrams for the latter devices at *V*_*GS*_ = 0 and *V*_*DS*_ = 0.5 V. (**f**) Transfer characteristics for the 12 nm-long channel FETs, optimized for HP processes. (**g**) Transfer characteristics for the 12-nm-long channel optimized for HD processes. All results have been obtained by mapping *E*_*on-site*_ from DFT. This figure was made using VMD [v1.9.1, https://www.ks.uiuc.edu/Research/vmd/vmd-1.9.1] and Matplotlib [v3.3.3, https://doi.org/10.5281/zenodo.4268928].

A relevant advantage of the method presented in this work is that it enables to model LHs with non-ideal interfaces. In the previous section we have seen the importance of mapping atomic interface details in a situation where small-gap semiconducting leads of 2L PtSe_2_ are attached to the larger-gap 1L channel. We now move to analyze a 4L-1L LH of PtSe_2_, where interfaces present the additional degree of freedom of allowing for smooth junctions in the form of slightly tilted 4L-1L interfaces (see Fig. 5a). As a first approximation we decided not to undertake a rigorous study of the actual structural stability of the exposed edges of the 4L regions. As for the 2L-1L structures, we have ensured Se terminations to minimize in-plane electric fields across the 1L region, and we have relaxed the positions of all edge Se atoms until residual forces were below 10^–3^ Ry/Bohr. Contrary to other state-of-the-art approaches, atomic-scale details on the channel-lead barrier can now be accounted for in the transport simulations, with the additional advantage of keeping the computational cost down to that required for a perfectly sharp junction.

All details regarding the construction and validation of the MLWF Hamiltonian for the 4L-1L PtSe_2_ LH are reported in the Supplementary Information. Hereafter we report on the performance prediction of a double-gated 4L-1L PtSe_2_ LH-FETs far from equilibrium, and the variations associated to the ab-initio modelling of the sharp and smooth interfaces. To this end we simulate the transfer characteristics of the device shown in Fig. 5b, with semi-infinite 4L leads and a 6.4 or 12.8-nm long 1L channel. Since the 4L system is metallic, transport is ambipolar. We apply a drain-to-source bias voltage *V*_*DS*_ = 0.5 V and scan over both positive and negative top/bottom gate voltages *V*_*GS*_, corresponding to electron and hole transport, respectively. For all devices, the top/bottom gates are long as the channel and the dielectric layers (namely SiO_2_) separating the channel from the gates have an equivalent oxide thickness of 0.5 nm.

The curves obtained for the 6.4 nm channel length are shown in Fig. 5c. The off-state current *I*_*off*_ in this case is always larger than 10^–4^ A m^−1^ for any applied *V*_*GS*_, due to intraband tunnelling which dominates transport across such a short channel, therefore we only assessed the performance of this particular device for HP applications. The *E*_*on-site*_ profiles and band diagrams at *V*_*GS*_ = 0 and *V*_*DS*_ = 0.5 V for the LH with 12.8-nm long channel are illustrated in Fig. 5d,e, for both sharp and smooth interfaces. The calculated transfer characteristics, optimized for both HP and HD applications, are shown in Fig. 5f,g. The main figures of merit, namely the SS and the *I*_*on*_*/I*_*off*_ ratio, for both HP and HD process optimizations are extracted and reported in Table 2, for all the modelled devices. We find that these do not differ significantly from the values obtained for the 2L-1L LH-FETs, reported in Table 1. Furthermore, we observe that a 3-nm smooth interface between 1 and 4L regions, rather than an atomically sharp one, does not alter the performance of LH-FETs significantly. This is especially true for the n-branch of the transfer characteristics, with only few differences in the p-branch for *V*_*GS*_ above a certain threshold, namely *V*_*GS*_ − *V*_*off*_ =  ~ − 0.2 V for HP and ~ − 0.4 V for HD optimizations, where current across the device with sharp junctions becomes slightly larger. We attribute this deviation to the fact that the *E*_*on-site*_ profiles in the devices with sharp or smooth interfaces are slightly different (see Supplementary Figure S4), yielding a Schottky barrier for holes which is 0.1 eV lower in the sharp case compared to the smooth case.

**Table 2:** Figures of merit for 4L-1L PtSe_2_ FETs. All figures of merit were obtained fixing the supply voltage *V*_*DS*_ = 0.5 V, and by mapping *E*_*on-site*_ from DFT. The IV curves from which the data were extracted are reported in Fig. 5.

	L [nm]	Branch	High-performance		Low-power/High-density	
			SS [ mV dec^−1^]	I_on_ / I_off_	SS [mV dec^−1^]	I_on_/I_off_
4L-1L-4L Sharp junction	6.4	p	71	1.5 × 10^4^	–	–
		n	75	2.0 × 10^4^	–	–
	12.8	p	67	1.6 × 10^4^	73	9.4 × 10^5^
		n	68	2.1 × 10^4^	68	6.9 × 10^5^
4L-1L-4L Smooth junction	6.4	p	76	1.0 × 10^4^	–	–
		n	75	1.8 × 10^4^	–	–
	12.8	p	71	1.0 × 10^4^	72	4.2 × 10^5^
		n	71	1.9 × 10^4^	68	6.2 × 10^5^

## Conclusion

We have presented a simulation study of FETs based on LHs of mono-multilayer PtSe_2_, based on a proposed multi-scale procedure which allows accurate *ab-initio* modelling of device interfaces.

As an application, we have explored various device configurations, namely with two and four layers in the multilayer regions, two different sub-15 nm channel lengths, as well as sharp or 3-nm smooth junction between 4 and 1L regions. All the results were obtained using a multiscale approach which consists in extracting the on-site energy from a DFT model of the LH and including it into a device Hamiltonian expressed in a MLWF basis, which is then used as input for the NanoTCAD ViDES device simulator^30^. This enables far-from-equilibrium transport simulations with *ab-initio* interface modelling, offering improved accuracy at a convenient computational cost.

We find that almost all devices, especially those with longer channels, yield a nearly optimal SS in the range 61–76 mV dec^−1^ and average *I*_*on*_/*I*_*off*_ ratios larger than 10^4^ for the HP case (larger than 10^5^ for the HD case). We also find that a modest smoothening of the junction between a 1L and a multilayer region of PtSe_2_ does not affect significantly the LH-FET performance.

Given the degree of ideality of our simulations (no electron–phonon scattering, nor defects or series resistances are considered) these results are to be considered as an upper limit for device performance. The ON currents of planar devices fall short of the requirements of the most recent version of the semiconductor technology Roadmap^28^. However, the roadmap focuses for the next decade on stacked channel devices, whereas here we have considered a single channel transistor. 2D materials are very well suited for stacked channel devices^2^ which provide a relatively straightforward way to achieve the ON current requirements, also considering the ON current reduction associated to the non ideality effects (e.g., finite contact resistance and scattering from phonons and defects in the channel) that we have not considered here.

From the methodological point of view, we have shown that correctly capturing charge displacements at interfaces is important when describing transport in p-type 2L-1L PtSe_2_ LH-FETs. Namely, holes accumulation in their proximity is reproduced, which has an important impact on the computed current–voltage characteristics, in terms of a significantly lower *I*_*on*_/*I*_*off*_ ratio and a higher tunnelling current in the sub-threshold regime. We have also demonstrated that the method allows to simulate heterostructures with non-ideal junctions, such as smooth ones between monolayer and multilayer crystals of the same material.

Despite its computational convenience, chemical details with this approach can only be accounted for indirectly, by means of variations in the ab-initio on-site potential profile. Its accuracy is not comparable to full DFT-based transport codes, which should always be adopted at least to provide an *a-priori* understanding of interface effects on transport for small device models. For instance, this might provide better insights on how the E_onsite_ profile might distort as a result of doping or applied biases far out of equilibrium. Reproducing such distortions requires a proper ab-initio simulation of the system out-of-equilibrium, and falls outside the scope of this work, for which capturing equilibrium band distortions around interfaces already represents an important improvement with respect to low-cost state-of-the-art transport models, where such effects are not taken into account.

We believe that the mapping procedure presented here will play a critical role in the context of devices based on fully monolayer LHs of different 2D materials^41–43^, or in any structure where intense dipoles, mixed stoichiometries or other chemistry-driven effects occur at the interfaces.

## Supplementary Information

Supplementary Information.

## References

[1] Novoselov KS, Mishchenko A, Carvalho A, Neto AHC (2016). 2D materials and van der Waals heterostructures. Science.

[2] Iannaccone G, Bonaccorso F, Colombo L, Fiori G (2018). Quantum engineering of transistors based on 2D materials heterostructures. Nat. Nanotechnol..

[3] Manzeli S, Ovchinnikov D, Pasquier D, Yazyev OV, Kis A (2017). 2D transition metal dichalcogenides. Nat. Rev. Mater..

[4] Yim C, Lee K, McEvoy N, O’Brien M, Riazimehr S, Berner NC, Cullen CP, Kotakoski J, Meyer JC, Lemme MC, Duesberg GS (2016). High-performance hybrid electronic devices from layered PtSe2 films grown at low temperature. ACS Nano.

[5] Ciarrocchi A, Avsar A, Ovchinnikov D, Kis A (2018). Thickness-modulated metal-to-semiconductor transformation in a transition metal dichalcogenide. Nat. Commun..

[6] Villaos RAB, Crisostomo CP, Huang ZQ, Huang SM, Padama AAB, Albao MA, Lin H, Chuang FC (2019). Thickness dependent electronic properties of Pt dichalcogenides. npj 2D Mater. Appl..

[7] Ansari L, Monaghan S, McEvoy N, Coileáin CÓ, Cullen CP, Lin J, Siris R, Stimpel-Lindner T, Burke KF, Mirabelli G, Duffy R, Caruso E, Nagle RE, Duesberg GS, Hurley PK, Gity F (2019). Quantum confinement-induced semimetal-to-semiconductor evolution in large-area ultra-thin PtSe2 films grown at 400 °C. npj 2D Mater. Appl..

[8] Ghorbani-Asl M, Kuc A, Miró P, Heine T (2016). A single-material logical junction based on 2D crystal PdS2. Adv. Mater..

[9] Li P, Li L, Zeng XC (2016). Tuning the electronic properties of monolayer and bilayer PtSe2 via strain engineering. J. Mater. Chem. C..

[10] Su TY, Medina H, Chen YZ, Wang SW, Lee SS, Shih YC, Chen CW, Kuo HC, Chuang FC, Chueh YL (2018). Phase-engineered PtSe2-layered films by a plasmaassisted selenization process toward All PtSe2-based field effect transistor to highly sensitive, flexible, and wide-spectrum photoresponse photodetectors. Small.

[11] Fiori G, Betti A, Bruzzone S, Iannaccone G (2012). Lateral graphene–hBCN heterostructures as a platform for fully two-dimensional transistors. ACS Nano.

[12] Marin EG, Marian D, Perucchini M, Fiori G, Iannaccone G (2020). Lateral heterostructure field-effect transistors based on two-dimensional material stacks with varying thickness and energy filtering source. ACS Nano.

[13] Chan, M., Assaderaghi, F., Parke, S. A., Yuen, S. S., Hu, C. & Ko, P. K. Recess channel structure for reducing source/drain series resistance in ultra-thin SOI MOSFETs. *Proc.**IEEE**Int.**SOI**Conf.* 172 (1993)

[14] Yang Y, Jang SK, Choi H, Xu J, Lee S (2019). Homogeneous platinum diselenide metal/semiconductor coplanar structure fabricated by selective thickness control. Nanoscale.

[15] Li L, Xiong K, Marsell RJ, Madjar A, Strandwitz NC, Hwang JCM, McEvoy N, McManus JB, Duesberg GS, Göritz A, Wietstruck M, Kaynak M (2018). Wafer-scale fabrication of recessed-channel PtSe2 MOSFETs with low contact resistance and improved gate control. IEEE Trans. Electron Dev..

[16] Xiong K, Li L, Marstell RJ, Madjar A, Strandwitz NC, Hwang JCM, Lin Z, Huang Y, Duan X, Göritz A, Wietstruck M, Kaynak M (2018). Improvement by channel recess of contact resistance and gate control of large-scale spin-coated MoS2 MOSFETs. IEEE Electron Device Lett..

[17] Jia J, Jang SK, Lai S, Xu J, Choi YJ, Park JH, Lee S (2015). Plasma-treated thickness-controlled two-dimensional black phosphorus and its electronic transport properties. ACS Nano.

[18] Liu X, Chen KS, Wells SA, Balla I, Zhu J, Wood JD, Hersam MC (2017). Scanning probe nanopatterning and layer-by-layer thinning of black phosphorus. Adv. Mater..

[19] Chen FW, Ilatikhameneh H, Ameen TA, Klimeck G, Rahman R (2017). Thickness engineered tunnel field-effect transistors based on phosphorene. IEEE Electron Device Lett..

[20] Kim S, Myeong G, Shin W, Lim H, Kim B, Jin T, Chang S, Watanabe K, Taniguchi T, Cho S (2020). Thickness-controlled black phosphorus tunnel field-effect transistor for low-power switches. Nat. Nanotechnol..

[21] Tung RT (2014). The physics and chemistry of the Schottky barrier height. Appl. Phys. Rev..

[22] Ávalos-Ovando O, Mastrogiuseppe D, Ulloa SE (2019). Lateral heterostructures and one-dimensional interfaces in 2D transition metal dichalcogenides. J. Phys. Condens. Matter.

[23] Bruzzone S, Iannaccone G, Marzari N, Fiori G (2014). An open-source multiscale framework for the simulation of nanoscale devices. IEEE Trans. Electron Devices.

[24] Szabó Á, Rhyner R, Luisier M (2015). Ab initio simulation of single and few-layer MoS2 transistors: effect of electron-phonon scattering. Phys. Rev. B.

[25] D’Amico P, Agapito L, Catellani A, Ruini A, Curtarolo S, Fornari M, Nardelli MB, Calzolari A (2016). Accurate ab-initio tight-binding Hamiltonians: Effective tools for electronic transport and optical spectroscopy from first principles. Phys. Rev. B.

[26] Marian D, Dib E, Cusati T, Marin EG, Fortunelli A, Iannaccone G, Fiori G (2017). Transistor concepts based on lateral heterostructures of metallic and semiconducting phases of MoS2. Phys. Rev. Appl..

[27] Szabó Á, Jain A, Parzefall M, Novotny L, Luisier M (2019). Electron transport through metal/MoS2 interfaces: edge- or area-dependent process?. Nano Lett..

[28] International Roadmap for Devices and Systems, 2020 edition, https://irds.ieee.org/editions/2020. Accessed June 2020

[29] Papior N, Lorente N, Frederiksen T, García A, Brandbyge M (2017). Improvements on non-equilibrium and transport Green function techniques: the next-generation transiesta. Comput. Phys. Commun..

[30] NANOTCAD VIDES code and documentation, http://vides.nanotcad.com. Accessed: June 2020

[31] Giannozzi P, Baroni S, Bonini N, Calandra M, Car R, Cavazzoni C, Ceresoli D, Chiarotti GL, Cococcioni M, Dabo I, Corso AD, de Gironcoli S, Fabris S, Fratesi G, Gebauer R, Gerstmann U, Gougoussis C, Kokalj A, Lazzeri M, Martin-Samos L, Marzari N, Mauri F, Mazzarello R, Paolini S, Pasquarello A, Paulatto L, Sbraccia C, Scandolo S, Sclauzero G, Seitsonen AP, Smogunov A, Umari P, Wentzcovitch RM (2009). Quantum Espresso: a modular and open source software project for quantum simulations of materials. J. Phys. Condens. Matter.

[32] Perdew JP, Burke K, Ernzerhof M (1996). Generalized gradient approximation made simple. Phys. Rev. Lett..

[33] Grimme S (2004). Accurate description of van der Waals complexes by density functional theory including empirical corrections. J. Comput. Chem..

[34] Bengtsson L (1999). Dipole correction for surface supercell calculations. Phys. Rev. B.

[35] Kandemir A, Akbali B, Kahraman Z, Badalov SV, Ozcan M, Iyikanat F, Sahin H (2018). Structural, electronic and phononic properties of PtSe2: from monolayer to bulk. Semicond. Sci. Technol..

[36] Furuseth S, Selte K, Kjekshus A (1965). Redetermined crystal structures of NiTe2, PdTe2, PtS2, PtSe2, and PtTe2. Acta Chem. Scand..

[37] Wang Y, Li L, Yao W, Song S, Sun JT, Pan J, Ren X, Li C, Okunishi E, Wang YQ, Wang E, Shao Y, Zhang YY, Yang HT, Schwier EF, Iwasawa H, Shimada K, Taniguchi M, Cheng Z, Zhou S, Du S, Pennycook SJ, Pantelides ST, Gao HJ (2015). Monolayer PtSe2, a new semiconducting transition-metal-dichalcogenide, epitaxially grown by direct seleni-zation of Pt. Nano Lett..

[38] Haastrup S, Strange M, Pandey M, Deilmann T, Schmidt PS, Hinsche NF, Gjerding MN, Torelli D, Larsen PM, Riis-Jensen AC (2018). The computational 2D materials database: high-throughput modeling and discovery of atomically thin crystals. 2D Materials.

[39] Mostofi AA, Yates JR, Pizzi G, Lee YS, Souza I, Vanderbilt D, Marzari N (2014). An updated version of WANNIER90: a tool for obtaining maximally-localised Wannier functions. Comput. Phys. Commun..

[40] Cusati T, Fiori G, Gahoi A, Passi V, Lemme MC, Fortunelli A, Iannaccone G (2017). Electrical properties of graphene-metal contacts. Sci Rep..

[41] Gong Y, Lin J, Wang X, Shi G, Lei S, Lin Z, Zou X, Ye G, Vajtai R, Yakobson BI, Terrones H, Terrones M, Tay BK, Lou J, Pantelides ST, Liu Z, Zhou W, Ajayan PM (2014). Vertical and in-plane heterostructures from WS2/MoS2 monolayers. Nat. Mater..

[42] Han Y, Li MY, Jung GS, Marsalis MA, Qin Z, Buehler MJ, Li LJ, Muller DA (2018). Sub-nanometre channels embedded in two-dimensional materials. Nat. Mater..

[43] Duan X, Wang C, Shaw JC, Cheng R, Chen Y, Li H, Wu X, Tang Y, Zhang Q, Pan A, Jiang J, Yu R, Huang Y, Duan X (2014). Lateral epitaxial growth of two-dimensional layered semiconductor heterojunctions. Nat. Nanotechnol..

