# Supplementary material for: Physical insights on transistors based on lateral heterostructures of monolayer and multilayer PtSe2 via Ab initio modelling of interfaces
Source: Sci Rep. 2021 Sep 16;11:18482. doi: 10.1038/s41598-021-98080-y (PMC8446074; doi:10.1038/s41598-021-98080-y)
Supplement: Supplementary file 1 — Supplementary Information. [file 41598_2021_98080_MOESM1_ESM.docx]

Supporting Information

Physical Insights On Transistors Based On Lateral Heterostructures Of Monolayer And Multilayer PtSe_2_ Via Ab Initio Modelling Of Interfaces

Gaetano Calogero ^a,b,*^, Damiano Marian ^a^, Enrique G. Marin ^c^, Gianluca Fiori ^a^ and Giuseppe Iannaccone ^a,^*

^a^ Dipartimento di Ingegneria dell'Informazione, Università di Pisa, Via Girolamo Caruso 16, 56122, Pisa, Italy

^b^ Consiglio Nazionale delle Ricerche, Istituto per le Microelettronica e Microsistemi, Z.I. VIII Strada 5, 95121 Catania, Italy
^c^ Dpto. Electronica, Facultad de Ciencias, Universidad de Granada, 18071, Granada, Spain

* Corresponding author

E-mails: [giuseppe.iannaccone@unipi.it](mailto:giuseppe.iannaccone@unipi.it) , [gaetano.calogero@imm.cnr.it](mailto:gaetano.calogero@imm.cnr.it)

Hamiltonian dimension

The device Hamiltonian built with the multi-scale procedure has a dimension *N_tot_ = N_c_ × N_s_*, where *N_c_* is the full length of the device in units of *a_x_* , and *N_s_* is the (uniform) dimension of each block in the matrix. The latter is in turn given by *N_s_* = *N_w_* *× (N_k_* /2 – 1) where *N_w_* is the number of Wannier Functions and *N_k_* is the number of $\boldsymbol{k}$-points used in the DFT and Wannier90 simulations along the periodic direction *y*. In all our calculations *N_w_* = 12 and *N_k_* = 12, therefore *N_s_* = 60.

In the grid for solving the Poisson equation, all the MLWFs within each of the *N_c_* elementary cells forming the device are grouped together to form a single device site, whose charge is the sum of all MLWF eigenstates for that cell. This is done to avoid charges very close one to each other, which could cause spurious behavior, and does not affect the NEGF part of the calculation.

Mobile and fixed carrier density profiles


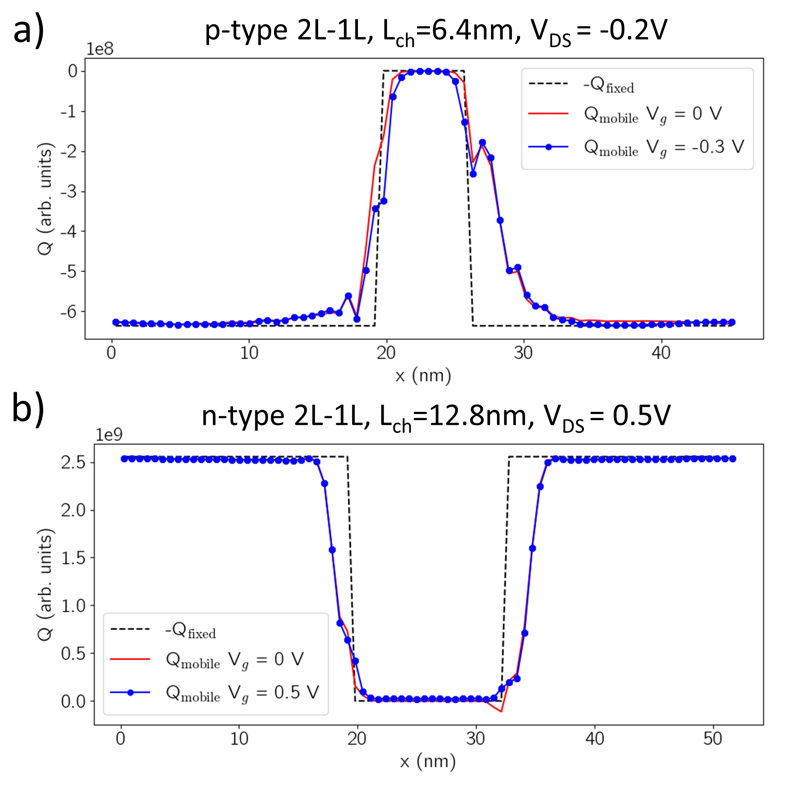


**Figure S1.** **Mobile and (minus) fixed** **carrier density profiles at different gate voltages in two representative 2L-1L PtSe_2_ LH-FETs** a) p-type with 6.4 nm long channel at V_DS_=-0.2V; b) n-type with 12.8 nm long channel at V_DS_=0.5V. Both panels show that fixed and mobile charges neutralize each other far from the channel, where Neumann boundary condition is assumed. This figure was made using Matplotlib [v3.3.3, https://doi.org/10.5281/zenodo.4268928].

Transfer characteristics of 2L-1L PtSe_2_ LH-FETs


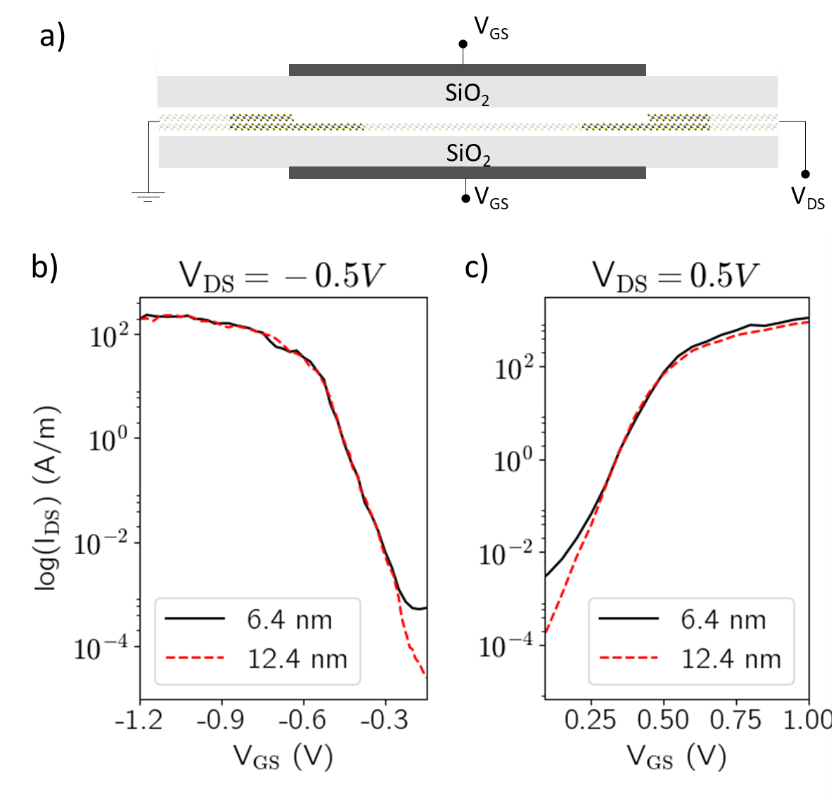


**Figure S2.** **Transfer characteristics of 2L-1L PtSe_2_ LH-FETs** a) Device double-gate configuration. b) Transfer characteristics for a 6.4 nm and a 12.8 nm long channel at *V_DS_* = -0.5 V c) Transfer characteristics at *V_DS_* = 0.5 V. This figure was made using VMD [v1.9.1, https://www.ks.uiuc.edu/Research/vmd/vmd-1.9.1] and Matplotlib [v3.3.3, https://doi.org/10.5281/zenodo.4268928].

DFT and MLWF modelling of 4L PtSe_2_

The unit cell and band structure for the 4L PtSe_2_ system are shown **Figure S3**a and S3b. We find very similar lattice parameters to the 2L, and a semimetallic electronic behaviour. Contrary to the 1L and 2L systems, for the 4L one “wannierization” (i.e., projection onto a MLWF basis set) within the considered energy window could not be achieved, due to difficulties in disentangling the large number of bands therein. The only wannierization that we managed to achieve required a large number of Wannier Functions (at least 88) to span a larger energy window, which however yields a Hamiltonian size difficult to manage. Therefore, as a first approximation, we decided to model the MLWF Hamiltonian of the 4L system using a simplified toy model. This was generated by wannierizing the 1L system on an energy window centered around an energy where the DOS is the same as the DOS obtained for the 4L system at its Fermi level, still using 12 Wannier Functions. This energy value should be thought of as the Fermi level *E_F_* in our 4L toy model. The toy model bands are shown in Figure S3c, while in Figure S3d we compare the toy model DOS with that of the actual 4L bulk system around the Fermi level. It can be seen that DOS away from the Fermi level is different in the two cases, but this does not affect significantly our devices simulations. We point out that another more technical reason for using a toy model for the 4L PtSe_2_ is that the implementation of the approach gains a lot in simplicity when the MLWF basis of the two junction component materials (i.e. the size of each block in the LH Hamiltonian) has the same dimension.


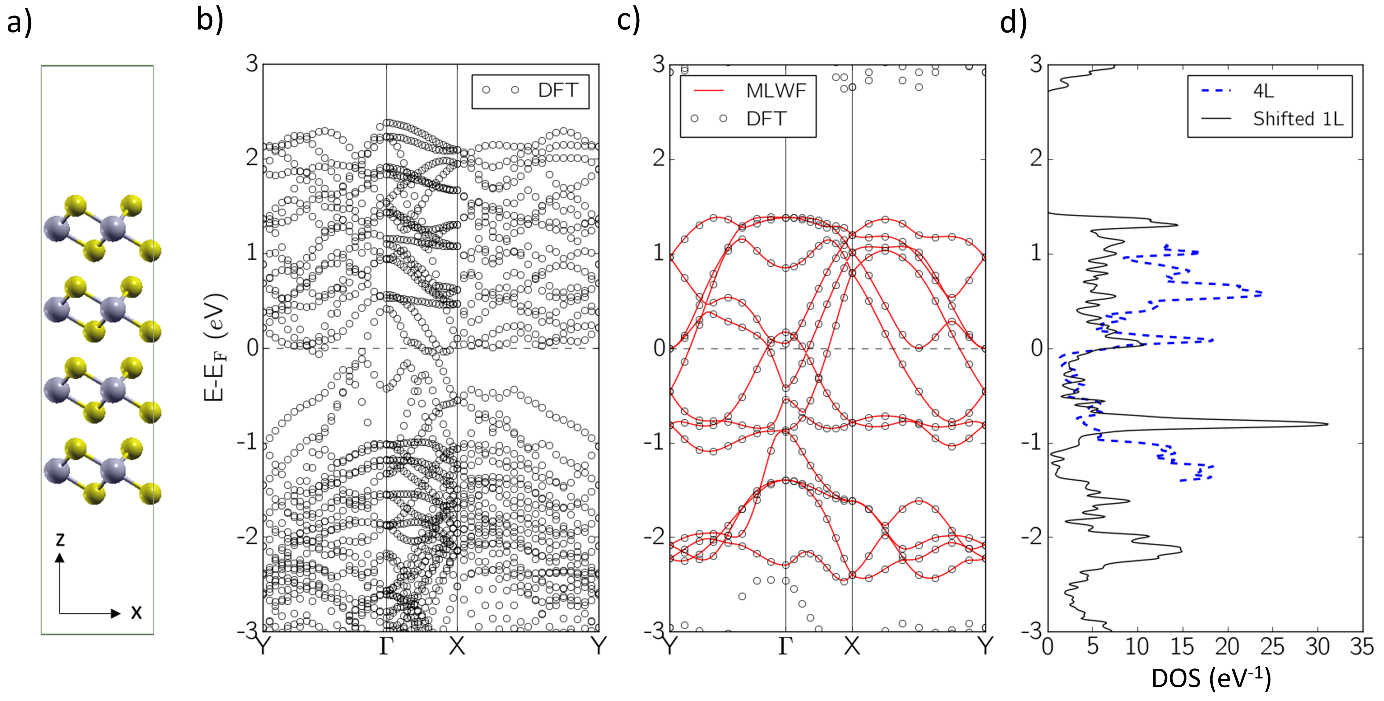


**Figure S3.** **DFT toy model for four-layer PtSe_2_.** a) Bulk unit cell. b) DFT bands of real 4L bulk system c) DFT and Wannier bands for the 4L toy model, created by rigidly shifting the 1L band-structure such that the DOS at *E=0* is equal to the DOS of the real bulk 4L system at its *E_F_*. d) DOS of the real 4L system (blue dashed) in comparison with the DOS for the toy model (black solid). This figure was made using VMD [v1.9.1, https://www.ks.uiuc.edu/Research/vmd/vmd-1.9.1] and Matplotlib [v3.3.3, https://doi.org/10.5281/zenodo.4268928].

Construction of the Hamiltonian for the 4L-1L PtSe_2_ LH

The 4L-1L-4L system was constructed similarly to the 2L-1L-2L, with either a 6.4 nm or 12.8 nm long 1L region, considering both a sharp and a 3 nm smooth junction. The final geometries are illustrated in **Figure S4**a and S4b. For the 4L-1L system the on-site energy extraction was done in the same way as for the 2L-1L-2L system, considering that for a metal such as 4L PtSe_2_ the Fermi level should be considered instead of the midgap level:

$E_{on-site} \left( x \right)\equiv\left\{ \begin{aligned} E_{F, 4L in HS} \left( x \right) x \in4L \mathrm{region} \mathrm{of} \mathrm{HS} \\ E_{midgap, 1L in HS} \left( x \right) x \in1L \mathrm{region} \mathrm{of} \mathrm{HS} \end{aligned} \right.$ (S1)

The results are shown in Figure S3c and S3d for the structures with sharp and smooth interfaces, respectively, using the DFT heterostructure Fermi level as reference zero energy. We plot the *E_on-site_* profiles (dotted lines) obtained after extracting the electrostatic potential at the three fixed ( *y_ref_, z_ref_* ) lines discussed in the main text, along with their average value (black line). The distance between points in each curve is *a_x_*. As expected, we find also here that *E_on-site_* along the heterostructure shows a ~ 0.4 eV peak in the 4L part of the junctions, as a direct consequence of charge redistribution. We also observe a slight tilt of the channel on-site energies towards the right interface for the sharp interface, which might be due to the slightly asymmetric terminations of the two exposed 4L edges. A slight deviation from zero can also be observed in the 4L regions far from the interfaces, probably due to the fact that the DFT supercell is not long enough to let the Fermi level in the 4L regions converge to that of an isolated 4L system. Nevertheless, this will not represent a limitation in a device simulator, where the only relevant information is the relative band alignment between 4L and 1L bands.

From the average curves in Figure S4c and S4d one can read the position of the Fermi level in the 4L regions of the heterostructure and deduce that of the conduction band edge in the 1L region. This is shown in Figure S4e and S4f for sharp and smooth cases, respectively. From these profiles the height of the Schottky barrier in the two LHs with sharp and smooth interfaces is estimated to be ~ 0.8 eV and ~ 0.7 eV, respectively.


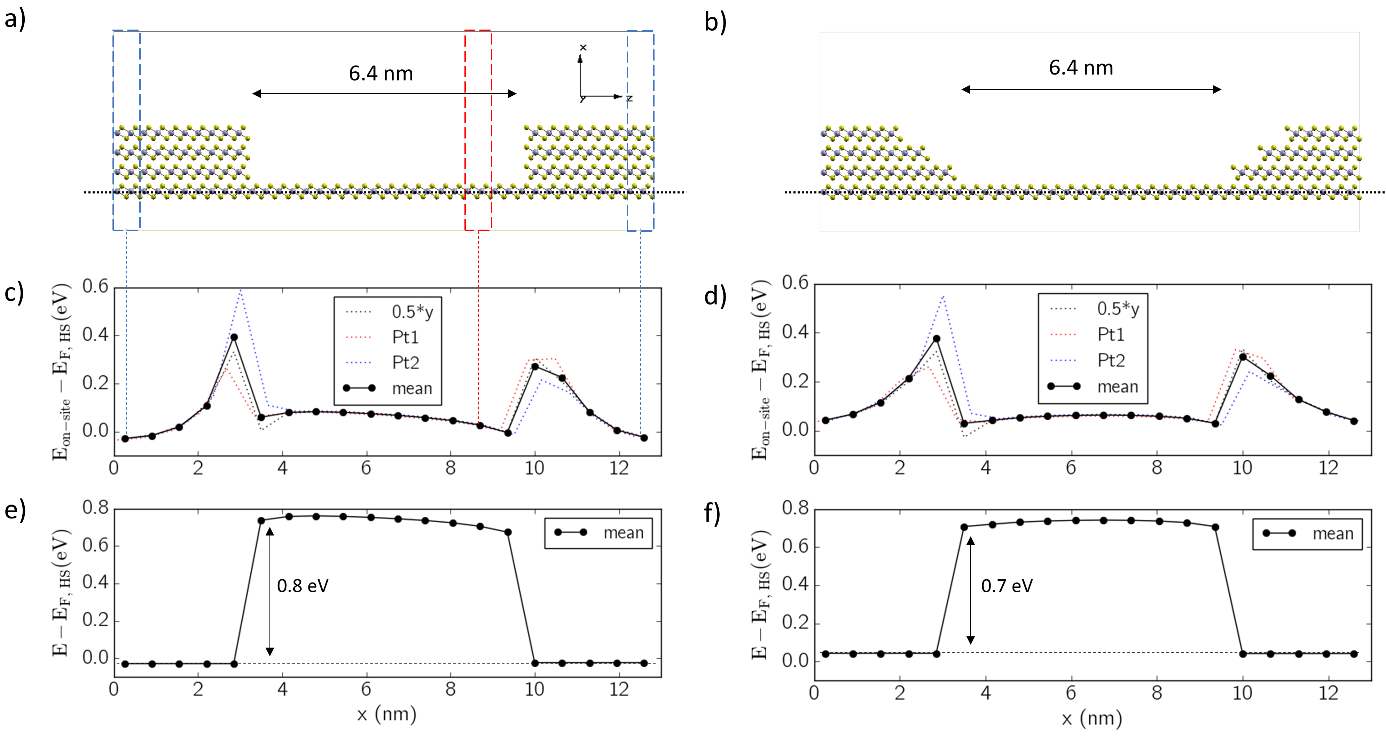


**Figure S4.** **DFT models of multilayer-monolayer PtSe_2_ heterostructures.** a-b) DFT supercells for heterostructure with sharp and smooth interfaces. The unit cells of bulk 4L and bulk 1L are shown as dashed blue and red boxes, respectively. c-d) Variation of the on-site energy *E_on-site_* along the device w.r.t. the equivalent isolated bulk fragments. Different dotted lines correspond to different reference lines at fixed *y* coordinate used to compare the electrostatic potential of the heterostructure to that of the bulk fragments, namely at that of “Pt1” and “Pt2” atoms indicated in Figure 2a, and at half the *y* cell vector. The average of these curves is also shown (solid black). e-f) Conduction band profile along the channel derived from the curves in c) and d). In the whole 4L regions the curves represent the Fermi level *E_F_*, corresponding to the minimum between left- and right-most potentials in c) and d). From these profiles the Schottky barrier height can be estimated. This figure was made using VMD [v1.9.1, https://www.ks.uiuc.edu/Research/vmd/vmd-1.9.1] and Matplotlib [v3.3.3, https://doi.org/10.5281/zenodo.4268928].
